# Implementing community-based health program in conflict settings: documenting experiences from the Central African Republic and South Sudan

**DOI:** 10.1186/s12913-023-09733-9

**Published:** 2023-07-08

**Authors:** Faiza Rab, Donya Razavi, Mariam KONE, Salim Sohani, Mekdes Assefa, Muhammad Haaris Tiwana, Rodolfo Rossi

**Affiliations:** 1 Department of Epidemiology and Biostatistics, Schulich School of Medicine and Dentistry, University of Western Ontario, London, ON Canada; 2 Health in Emergencies, Canadian Red Cross, Ottawa, ON Canada; 3 Health in International Long-Term Programming, Canadian Red Cross, London, Canada; 4 Department of Health, Aging and Society, McMaster University, Hamilton, ON Canada; 5 Masters of Public Health , University of Western Ontario, London, ON Canada; 6 Health Unit, International Committee of the Red Cross, Geneva, Switzerland

**Keywords:** Community based health services, Reproductive Maternal Neonatal Child and Adolescent health services, Armed conflict, Protracted conflict, Agile and responsive programing, Community engagement, The Central African Republic, South Sudan, RMNCAH

## Abstract

**Background:**

The delivery of quality healthcare for women and children in conflict-affected settings remains a challenge that cannot be mitigated unless global health policymakers and implementers find an effective modality in these contexts. The International Committee of the Red Cross (ICRC) and the Canadian Red Cross (CRC) used an integrated public health approach to pilot a program for delivering community-based health services in the Central African Republic (CAR) and South Sudan in partnership with National Red Cross Societies in both countries. This study explored the feasibility, barriers, and strategies for context-specific agile programming in armed conflict affected settings.

**Methods:**

A qualitative study design with key informant interviews and focus group discussions using purposive sampling was used for this study. Focus groups with community health workers/volunteers, community elders, men, women, and adolescents in the community and key informant interviews with program implementers were conducted in CAR and South Sudan. Data were analyzed by two independent researchers using a content analysis approach.

**Results:**

In total, 15 focus groups and 16 key informant interviews were conducted, and a total of 169 people participated in the study. The feasibility of service delivery in armed conflict settings depends on well-defined and clear messaging, community inclusiveness and a localized plan for delivery of services. Security and knowledge gaps, including language barriers and gaps in literacy negatively impacted service delivery. Empowering women and adolescents and providing context-specific resources can mitigate some barriers. Community engagement, collaboration and negotiating safe passage, comprehensive delivery of services and continued training were key strategies identified for agile programming in conflict settings.

**Conclusion:**

Using an integrative community-based approach to health service delivery in CAR and South Sudan is feasible for humanitarian organizations operating in conflict-affected areas. For agile, and responsive implementation of health services in conflict-affected settings, decision-makers should focus on effectively engaging communities, bridge inequities through the engagement of vulnerable groups, collaborate and negotiate for safe passage for delivery of services, keep logistical and resource constraints in consideration and contextualize service delivery with the support of local actors.

**Supplementary Information:**

The online version contains supplementary material available at 10.1186/s12913-023-09733-9.

## Introduction

Over the past two decades, substantial improvements have been made in maternal and child health, with considerable reductions in global maternal and childhood mortality [1, 2]. Despite the progress made in maternal and child healthcare, gaps in access and delivery of quality healthcare for women and children in conflict-affected settings remain a significant challenge [1, 3, 4]. Insecurity, service disruptions, destruction of crucial infrastructure, population displacements and competing priorities hamper the implementation of Reproductive, Maternal, Neonatal, Child and Adolescent Health (RMNCAH) services essential to reduce maternal, neonatal, and child mortality and morbidity [4]. Gaps in RMNCAH services in fragile and conflict-affected areas often stem from fragmented services and lack of continuity in the delivery of essential health services, and inefficient and often non-existent referral systems in these contexts [5, 6]. A reduction in maternal, neonatal and childhood mortality and morbidity to improve health and wellbeing of the most vulnerable communities is not possible unless the global health policy makers and implementers find an effective modality in conflict affected contexts [7, 8]. Despite a plethora of well-tested community-based interventions being available for low- and middle-income countries (LMICs), [9–11] these cannot be simply extrapolated in the conflict context since the challenges of insecurity and restriction can significantly hamper the access and delivery of services.

### Integrated public health approach to meet the needs of populations in conflict-settings

With a history of over 150 years of assisting the vulnerable populations during war and conflict, the International Committee of the Red Cross (ICRC) works in several areas related to protection during conflict, including health.^1^ The ICRC Health Strategy (2020–2023) pledges to provide high-quality healthcare services through an integrated public health approach to address the unmet needs of those impacted by protracted conflict [12]. The integrated public health approach ensures a continuum of care across service delivery at various stages of acute and protracted armed conflicts [12]. The ICRC collaborated with the Canadian Red Cross (CRC) in jointly developing a technical, institutional framework for a program called an Advanced Partnerships in Health (APiH),^2^ using the integrated public health approach, focused on RMNCAH. The integrated public health approach encompasses a recognition of the importance of proximity of provision of care to the victims of conflict, developing approaches and strategies to delivery of health services based on continuum of care from the community to the facility level. The approach integrates ICRC’s strategic objectives of ensuring continuum of care from first aid through primary care to secondary and tertiary healthcare in armed conflict settings; ensuring highest quality of healthcare contextualized to the conflict settings; responding to new and emerging health needs of people in conflict settings; enhanced integration of health services with other ICRC activities ^2^.

### The Advanced Partnership in Health (APiH) framework

**Fig. 1:**
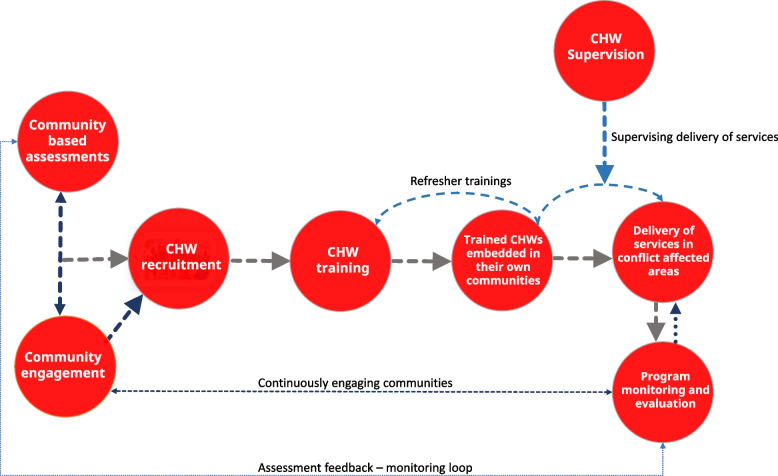
Overview of the process of delivering community based RMNCAH health programs in armed conflict settings

Through the APiH framework, the aim is to establish a multi-year collaboration between the ICRC, CRC, and the in-country Host Red Cross/Red Crescent National Societies (HNS), a unique program based on co-creation, adaptation or adoption of globally acceptable technical standards in the community-based RMNCAH in conflict settings. The application of the APiH framework aims to enhance the continuum of care for women and children from household and community levels and access to primary, secondary, and tertiary care through joint service delivery. The package of health services, identified through the APiH framework, includes basic or essential healthcare services such as antenatal, perinatal, and postnatal care, identifying and treating common childhood illnesses, and identifying referral pathways for more complex healthcare needs. The framework focuses on an inclusive, localized, and contextualized model for health service delivery and provides detailed guidance on core activities to be delivered through all programs, and contextual activities to be defined through community needs assessment before program implementation. Core and contextual activities at the primary and secondary health care levels are defined in detail under three areas in the APiH technical framework. *1. Educational/promotional*: examples – counselling and family planning promotion (core at primary level), promotion of benefits kangaroo mother care (core at secondary level), awareness raising on safe abortion (contextual at primary level), promotion of use of maternity waiting homes (contextual at secondary level). 2**.***Preventive care and commodities*: examples – provision of folic acid to pregnant women (core at primary care level), screening of gestational diabetes (contextual at primary care level), in ward immunization catch up (core at secondary level), long lasting insecticide treated bed nets (contextual at secondary level). *3. Risk, case identification and referral/curative care:* examples – management of uncomplicated abortion and treatment of anemia (core at primary care level), STI screening and prophylactic treatment for co-wives (contextual at primary care level), blood transfusions for severe anemia and post-partum hemorrhage (core at secondary care), care of premature neonates using incubators (contextual secondary). Figure 1 describes the process of delivering community-based RMNCAH health programs in armed conflict settings, using the APiH framework.

### APiH pilot programs

Between 2019 to 2021, the APiH framework was piloted in two, armed conflict-affected, countries with low access to RMNCAH services; the Central African Republic (CAR) and South Sudan. The two countries were chosen because they have been affected by protracted conflict for decades. According to the World Health Organization (WHO), CAR and South Sudan have some of the worst health indicators in the world [13]. Multiple crises have adversely impacted the health system in CAR, which has one of the highest mortality rates in the world, with latest neonatal, infant, and under 5 mortalities at 38.7, 77.5 and 103 per 1000 live births respectively, and maternal mortality rates of 882 per 100 000 live births [14]. The condition is even worse in conflict affected areas with crude mortality rate (CMR) of 1.57 /10,000/day [15]. A recent South Sudan also has some of the worst health outcome indicators, with a maternal mortality ratio at 789 per 100,000 live births, and neonatal, infant and under-five mortality rates are 40.2, 63.3 and 97.8 per 1000 live births, respectively [16].

To implement the APiH framework, the Health in Emergencies (HiE) unit at the CRC and ICRC Health Department in Geneva worked with the delegations and Host National (Red Cross/Red Crescent) Societies (HNS) in CAR and South Sudan. A program to deliver community-based health services for women, children and adolescents in conflict-affected settings was co-designed with inputs from the national red cross societies in both countries. These pilot projects focused on testing implementation modalities of the community-based RMNCAH services to increase the reach, complement, and strengthen the continuum of care of the existing ICRC-supported primary health care programs. The program implementation started in both countries with the APIH’s core package of services. There were similarities in core activities implemented in both countries including health promotion both at facility and community levels; community-based surveillance of specific RMNCAH events (maternal death, neonatal death and home birth) as well as unusual events of public health importance (such as clusters of unexplained illness or deaths, population movement, etc.); health promotion focusing on RMNCAH issues (safe motherhood, family planning, nutrition including malnutrition cases, immunization including unvaccinated children referrals, hygiene etc.); distribution of preventive commodities such as condoms, clean delivery kits; case identification and referrals (high risk pregnancy, pregnant women with danger signs, children with danger signs); implemented by trained, equipped, and supported community-based health workers/volunteers’ networks.

#### APiH pilot program in the Central African Republic

In CAR, the Nana Grizibi region, specifically the Ouandaogo and Grava distrcts were selected for program implementation, which started in November 2020. Community consultations and engagements in CAR were arranged through extensive community mobilization by the Central African Republic Red Cross Society (CRCA) and took place in villages where a rapid scan of health needs was previously completed. From November 2020 to August 2022, fifty one meetings (31 in Ouandago and 20 in Grevai) took place with participants from 68 villages (participation from 39 villages in Ouandago and from 29 villages in Grevai) to assess the health needs of the local populations. In total 175 Community Health Workers (CHWs) and six supervisors were identified by the communities. In addition to the core activities the contextual activities in the later phase of the program included tracing of defaulters from routine immunization; nutrition programs and the establishment of a transportation system for community-based referrals. Additionally, the project in CAR included for the scale up phase, implementation of community-based epidemic preparedness and control activities, school health clubs (focusing on hygiene promotion and adolescents’ sexual reproductive health issues) as well as an innovative approach in malnutrition screening in children, namely the “MUAC Maman” (training mothers to identify malnutrition in their children using the MUAC measurement and self-refer).

#### APiH pilot program in South Sudan

In South Sudan, the project implementation started in July of 2019 in the Ngo Ku and Ngo Dakala in the Wau region. In South Sudan, chiefs and village elders were directly consulted. At this point planned activities were explained to them, and their permissions were sought to engage with the community. Consultations took place in local Basic Health Centres. From July 2020 to August 2022, twelve meetings were held with the village elders, 73 BHWs^3^ and 7 supervisors were identified and trained. In addition to the core activities, the program in South Sudan included the implementation of the Integrated disease surveillance and Response by the community health workers as per national guidelines in the later phases of program delivery.

Implementing the APiH framework was a unique opportunity to apply an integrated model of health services delivery in fragile and conflict-affected settings. This study examines the perspectives of program implementers and the community on the feasibility of using the integrated public health approach in the delivery of health services in conflict settings. Furthermore, the study explored the challenges and opportunities to improve health service delivery to women and children caught in protracted conflict.

## Methods

The study aimed to understand the feasibility of implementing community-based RMNCAH services in contexts affected by armed conflict. Researchers and program implementers at ICRC and CRC as well as HNS in CAR and South Sudan, were consulted to define the scope and research questions through a collaborative process. The objectives were designed to understand the implementation modalities in contexts affected by armed conflict settings through:

1. Exploring the perspectives of program implementers and community on feasibility of delivering community-based RMNCAH services
2. Identifying the barriers to delivering community-based RMNCAH services
3. Understanding strategies for context-responsive and agile and responsive program implementation in delivering health services

### Study design

A qualitative study design with key informant interviews (KII) and focus group discussions (FGD) was used for this study. The study was conducted in four districts where the APiH pilot programs were underway, two in CAR (Ouandaogo, Grevai) and two in South Sudan (Ngo Dakala & Ngo KU).

### Data collection

A purposive sampling methodology was used for data collection to maximize perspectives. Open-ended and semi-structured Interview guides were used to conduct KIIs with program implementers and FGDs with CHWs/volunteers, community elders, men, women, and adolescents in the community (KII and FGD guides are available as supplement 1). Data collection tools were developed based on the study objectives and were tested and modified through field consultations for the feasibility of use.

Program implementers in the field, known to the technical teams at ICRC and CRC were approached for the KIIs. The CRC research team collected KII data through Zoom and Teams video conferencing. FR and DR conducted KII in English with the South Sudanese participants MK conducted KII in French with the CAR participants. French data was translated into English and transcribed by research assistants and program officers at CRC.

Participants for the FGDs were identified by the field team and consisted of village elders, leaders, men, women, and adolescent already engaged with the field team in the implementation of the program. FGDs were conducted by experienced health field staff, experienced in FGDs. The health field teams supported participant recruitment, data collection (conducting focus groups), translations, and transcription of the data for the focus groups. COVID-19 restricted travel to the field for the team conducing the research, therefore, to ensure that the quality and integrity of data collection process the research team conducted ‘Training of Trainers’ (ToT) workshops with the CAR (French) and South Sudan (English) field teams. The ToTs covered how to prepare, conduct, and facilitate a focus group and the necessary materials required to conduct FGDs. The training included training on participant recruitment including consent and assent procedures, note taking, transcribing the audio recordings, handling data and data transportation. The training also included a psychological first aid (PFA) component that outlined the scope of PFA and emphasized the importance of self-care, how to identify a participant in distress, and how to provide PFA to participants when needed. The FGDs were conducted in local languages and the data notes were translated, transcribed and transferred to researchers at CRC for analysis.

### Data analysis

Data were analyzed using a content analysis approach [17]. Two researchers, FR and DR, developed a data extraction tool for each of the three study objectives (perspectives on feasibility, barriers to delivery of services and strategies to improve agility and responsiveness). FR and DR analyzed KII and FGD transcripts and notes to develop codes. The codes were discussed by FR and DR and converged into categories and themes where appropriate. The analysis was then discussed with the research team, and engaged in consensus building around the relevant codes, categories, and themes.

### Ethics approval

The study received approval (DP_DR 21/00011/ESV/abg) from the ICRC Ethics Review Board. The complete study protocol was shared with the Ministries of Health in South Sudan and CAR and approved by host Red Cross/Red Crescent National Societies in both countries. Written informed consent was obtained from each study participant before initiating each key informant interview. Verbal informed consent was obtained from each participant before initiating the study. The study procedures and methods were conducted in accordance with the ethical principals and guidance in the World Medical Association Declaration of Helsinki.

## Results

Data collection took place between May 1^st^ and June 6^th^, 2021. Fifteen KII and 16 FGDs were conducted in the two countries. A total of 169 people participated in this study (Table 1).

**Table 1:** Role and gender distribution of the participants

		**South Sudan**		**Central African Republic**	
**Key informant interviews**^a^	**Total participants = 15**	**Total participants = 7**		**Total participants = 8**	
		Males	Females^b^	Males	Females
	MoH	2	0	1	0
	ICRC	2	0	1	2
	HNS	1	1	2	0
	CRC/ICRC delegate in the field	1	0	2	0
**Focus groups (FG)**	**Total groups = 16** **Total participants = 154**	**Groups = 12** **Participants = 114**		**Groups = 4** **Participants = 40**	
	CHW/volunteers	# FG: 2 Total number of participants (all groups combined): 20 Type of group: Mixed (male and female) Catchment areas: Ngo Dakala (1), Ngo Ku (1) Female participants: 6 Male participants: 14		# FG: 1 # Participants: 10 Type of group: Males only Catchment areas: Ouandaogo	#FG: 1 # Participants: 10 Type of group: Females only Catchment areas: Ouandaogo
	Community leaders/ elders	# FG: 2 Total number of participants (all groups combined): 20 Type of group: Mixed (male and female) Catchment areas: Ngo Dakala (1), Ngo Ku (1) Female participants: 5 Male participants: 15		# FG: 1 # Participants: 10 Type of group: Males only Catchment areas: Ouandaogo	# FG: 1 # Participants: 10 Type of group: Males only Catchment areas: Ouandaogo
	Community members (men, women, and adolescents)	# FG: 4 Total number of participants (all groups combined): 39 Type of group: Males only Catchment areas: Ngo Dakala (2), Ngo Ku (2) Adult men: 2 groups, 19 participants Adolescent boys: 2 groups, 20 participants	# FG: 4 Total number of participants (all groups combined): 35 Type of group: Females only Catchment areas: Ngo Dakala (2), Ngo Ku (2) Adult women: 2 groups, 15 participants Adolescent girls: 2 groups, 20 participants		

^a^*MoH* Ministry of Health, *ICRC* International Committee of the Red Cross, *HNS* Host National Society, *CRC* Canadian Red Cross

^b^Preference was given to female participants, limited in recruiting female participants for KII as the sampling pool for females in leadership position was limited

In the following section, the identified themes have been organized according to the three study objectives: 1. Perspectives on feasibility of delivering community-based RMNCAH services in armed conflict, 2. Barriers to the delivery of RMNCAH services, and 3. Strategies to improve agility and responsiveness for program delivery in armed conflict settings. The details of how the themes were coded are presented in Supplement 2.

## Perspectives on feasibility of delivering community-based RMNCAH services in armed conflict

The analysis of KII and FGDs showed that the implementers engaged the tribal chiefs, community leaders, elders, beneficiary groups, including men and women in both countries, prior to program implementation. The KII and FGD analysis provided further insights regarding factors contributing to feasibility of delivering health services in armed conflict affected areas. Engaging community elders, who were considered ‘gate keepers’ to the community, emerged as an important consideration throughout the implementation process, underpinning the importance of gaining community ‘trust,’ for entry into the community, to improve buy in by the community and to identify and retain CHWs. Building trust was especially important in armed conflict settings where communities were forced to move into the bush.

Facilitating the process of program implementation was grouped under three codes which were identified as the three themes linked to factors contributing to feasibility of service delivery in armed conflict settings: (i) well-defined and clear messaging regarding the available services, (ii) inclusive participation for community consultations and (iii) delivering services by someone from the community.

### Well-defined and clear messaging regarding the available services

The KII from both countries identified that literacy rates were low in their countries. Because of low literacy levels in their countries, approaches that clearly provide key health messages through easy-to-understand materials, such as pictograms and simple messages would help CHWs conveniently deliver the message with communities being more receptive to simple messages. *“Literacy is an issue overall in South Sudan. Some can read and write, and some can understand if it’s explained to them and carry out their activities. Some are bit educated some not. Those who are not educated but can get proper explanation and can bring the message they are ok- The key thing is to understand health issues, they will need to understand cases and can manage using tools at community level”-* Program implementer, South Sudan.

The elders, community leaders and program implementers identified well defined, clear messaging in local languages to be an effective way to gain community trust and buy in and easily deliver services. *“If clear message is delivered and they understand the situation and they know what is going to take place and they will accept.—once they understand it by implementing in the project”-* Program implementer, South Sudan.

### Inclusive participation for community consultations

The participants emphasized the importance of inclusive participation to ensure that the program is accepted by the community. Although the participants overwhelmingly endorsed the importance of engaging with community leaders and elders. *“…it is not a problem to engage village elders because they have more and more experience and they have so much more information than adolescents”*—Adolescent boys group, South Sudan.

Other community members, especially women and adolescent also wanted to ensure that their voices are incorporated in program planning and implementation. In our focus group discussions with adolescent boys and girls, some were dissatisfied with engaging just the elderly and the need of engaging more with adolescents was strongly recommended to also bring their perspectives to the table. *“…for me the problem in engaging elder because – they may not [be] qualify [ied] person to deliver the information or have problem in his eyes or something like this and you [know] in the earlier time there was no education”-* Adolescent boys group, South Sudan. *“..train us in the delivery of health services”-* Adolescent girls group, South Sudan.

Describing the significance of consultations, a male CHW from CAR started that *“.. identifying the needs of youths was important for the development of the village.”*

### Delivering services by someone from the community

Commitment to stay with the community during the conflict was identified as key to selecting CHW volunteers. One key informant from CAR emphasized that the CHWs must be native in describing the eligibility criteria for CHW as: “*They have to be native to the village or the neighbourhood, and they cannot be insolent or insulting. Must not be minors but must be men and women who can read/write and are respected. Volunteers in the community elect people and send a list [volunteers from each village are selected from the list]”-* Description of eligibility criteria for CHW recruitment by a Key Informant in CAR*.*

Access to communities is often cut-off during the conflict. Having native and locally available trained CHWs will help support communities when there are issues with access. *“…we need to be engaged because we need for us health workers from women and from youth and from boys and girls in our community to learn about the work for the next time these people cannot come around [because of war] they [CHWs living in community] will do it”-* Womens group, South Sudan.

## Barriers to implementation of community-based RMNCAH services in conflict-affected areas

**Fig. 2:**
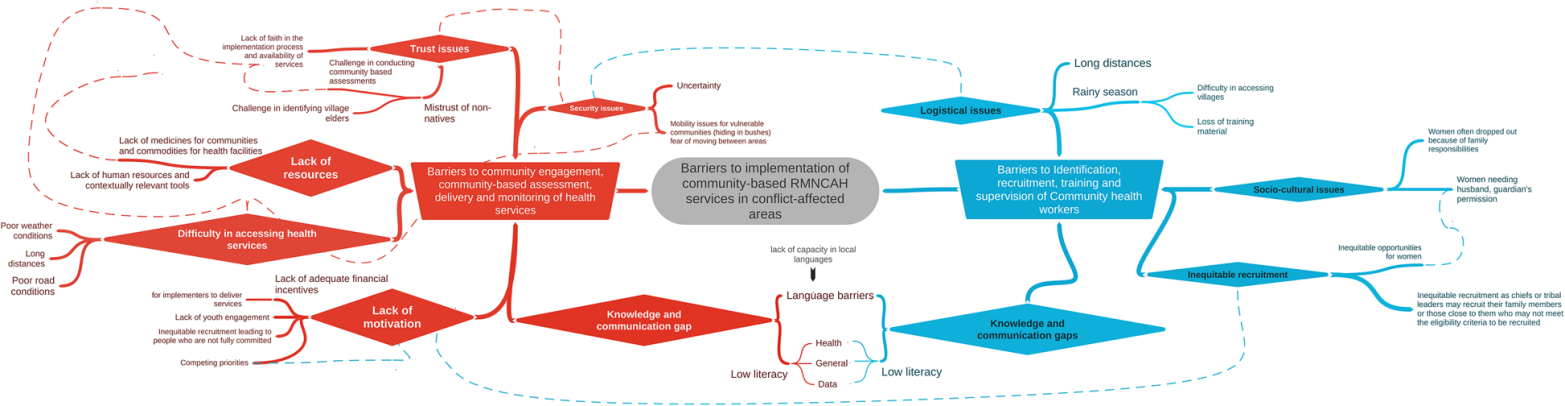
Themes, sub-themes and interconnectedness of barriers to implementation of RMNCAH services (Fig. 2 shows the overarching themes (Boxes-rectangle), themes (boxes-diamond) and sub-themes (nodes stemming from diamond boxes) related to barriers in implementing RMNCAH services. Barriers were grouped under two overarching themes:: i. barriers to community engagement, community-based assessments, delivery and monitoring of health services, ii. barriers to identification, recruitment, training, and supervision of CHWs. Sub-themes and themes linked with the overarching themes are presented as solid lines in the figure. There were several themes and sub-themes that were interconnected within and across themes, the interconnectedness is represented by dashed lines.)

Barriers to implementation of community-based RMNCAH services in conflict-affected areas were identified through analysis of KII and FGDs. These barriers were grouped under two overarching themes: i. barriers to community engagement, community-based assessments, delivery and monitoring of health services, and ii. barriers to identification, recruitment, training, and supervision of CHWs. Within these overarching themes, we identified categories of barriers that we labelled “themes”. Several codes contributed to the categories and were labelled as “sub-themes” in Fig. 2. Many of the themes and sub-themes were found to be interconnected. These relationships are presented in Fig. 2.

### Barriers to community engagement, community-based assessments, delivery and monitoring of health services

Similar barriers affected the program implementation process including community engagement, community-based assessments, delivery and monitoring of health services and were grouped as themes. These themes were security related issues, trust issues, lack of resources, difficulties in accessing health services, lack of motivation, and knowledge and communication gaps. Sub-themes (supplement 2) were interconnected (Fig. 2) and are presented in the explanation of the themes in this section.

#### Security related issues

*P*ertaining to the threat of acute conflict, uncertainty amidst protracted conflict, and physical barriers between government sectors and areas controlled by armed groups, emerged as a significant hindrance to all aspects of program implementation.

*“In our context the biggest challenge is security, I don’t think we have a bigger challenge than that”*- Program Implementer, Central African Republic.

The uncertainty linked with insecurity is a challenge to deliver health services in conflict affected areas.

*“Insecurity for the health delivery in conflict settings, which cause healthcare [workers] [to be] in danger for volunteers and CHWs and even delivery of medicines or stop of humanitarian services to reach the community and the vulnerable ones”* – Community Health Workers group, Central African Republic.

*“..insecurity in the village cannot let the delivery of the health services go on because the enemy may block the way for their benefit or may be if they brought for us some services like soap or drug they[we] use to take it from them”-* Men’s group, South Sudan.

#### Trust issues

Some focus group participants voiced a lack of faith in the implementation process, especially the process of assessments in the past, with little effort being put into implementation post assessments. *“We have participated in this kind of assessment many times and there is need to follow up on the assessment results for the success of the community members” –* Community leaders group South Sudan. Additionally, lack of availability of services also erodes trust in communities. *“When you give awareness and send them for services, and then if the person goes, then if the service is not available it affects trust”* – Program implementer, South Sudan.

Some participants also identified a mistrust towards those not from the community or non-natives, which hinders the process of properly identifying village elders further complicating the process of community assessments and service delivery.

#### Lack of resources

Identified as lack of medicines, human resources, referral pathways and contextually relevant tools, serve as barrier to program implementation. This lack of availability of resources also effects trust by the community as identified above by the community leader’s group and program implementer from South Sudan, resonated as a barrier in CAR, where a male CHW described *“ … lack of medicines, destruction of post offices or health centres, it’s enormous…”* challenge in delivery of services.

#### Difficulties in accessing health services

Poor weather (especially during rainy season) and road conditions, along with long distances result in difficulties for the communities to engage with the program implementers and access health services. *“We ask for waterproofs, means of transportation, bicycles to help pregnant women and for our travels to the health center”* – Male CHW, CAR. The vulnerable in the community, including women and children are most affected due to mobility related issues stemming from weather and infrastructure as well as insecurity*.*

#### Lack of motivation

Competing priorities for CHWs as they are volunteers and often need to find alternative sources of income to support their families demotivates potential CHWs to volunteer for recruitment, or if they are recruited they are not motivated to perform to the best of their abilities. This also contributes to barriers in recruitment of CHWs discussed under theme 2.2. *“..someone having responsibility at home they need to go for long period time. People are dependent on others to get daily meal so that would be difficult”-* Program implementer, South Sudan.

Other causes for lack of motivation identified by the participants were lack of youth engagement and delays in delivery of health services or implementation of programs.

#### Knowledge and communication gaps

Pertaining to language barriers, health literacy and miscommunications about available services. Language barriers were a consistent sub-theme for barriers across the implementation process. In describing these challenges two community leaders from CAR stated that *“ Some of our female colleagues have not been to school, it’s hard to understand” and “If we are given images, it would be easy as this is the beginning and we can adapt.”*

### Barriers to identification, recruitment, training, and supervision of CHWs

Similar barriers affected identification, recruitment, training, and supervision of CHWs and were grouped as themes. These themes were socio-cultural issues, inadequate recruitment, logistical issues, knowledge, and communication gaps. Sub-themes (supplement 2) were interconnected (Fig. 2) and are presented in the explanation of the themes in this section. The socio-cultural issues adversely impacted women as they women often dropped out of the CHW training or were not able to be recruited because of competing family responsibilities. While discussing women’s participation as CHWs, a program implementer from South Sudan stated that *“Approval from husbands needed, husbands can refuse.”**** I***nadequate participation by women also contributed to inadequate overall recruitment and lack of motivation for CHWs to deliver services.

The CHWs and supervisors also suffered from logistical challenges. Long distances and inadequate supplies during rainy season inhibit access to communities and can result in loss of training materials – *“when they go to conduct activities they ask about boots, rain coat, food.”* – Program Implementer, South Sudan. These issues further complicate delivery of services by contributing to uncertainty. *“Handling materials is a challenge in rainy season as their materials can get wet.”* – Program Implementer South Sudan.

Knowledge and communication gaps pertained to lack of overall literacy, as many of the identified CHWs in the past did not have school education, and were not able to read or write either. *“Most of them they don’t write. We need to continually recap to make them remember what they have.”* Program Implementer South Sudan. Lack of data literacy and miscommunications about available services and budget allocation were also identified as barriers along with language being a barrier for many CHWs during the training processes. *“Language barriers can be an issue, that is a big one.”*—Program Implementer South Sudan.

## Strategies for context specific agile and responsive programing in conflict settings

Our participants identified several solutions to overcome the barriers and strategies to improve context specific health programming. These strategies were organized under eight themes designed to support program implementation throughout the program cycle, especially in armed conflict contexts.

### Community engagement and leadership

Engaging chiefs, elders, community leaders, and relevant stakeholders was identified as the most important strategy to ensure community responsiveness, especially in conflict settings. *“Must identify the key actors in armed conflicts and understand their role and whether it is possible to engage them” –* Key informant, CAR.

*“Chiefs were there [community engagement and assessment sessions] so that provide legitimacy” – Key informant, South Sudan.*

Engagement of the communities often occurred through engaging elders and chiefs to ensure that community partners understand the project well and support its rollout in communities. *“..once they [community leaders] understand it by implementing in the project, this is where you justify [your presence in the community]” – *Key informant, South Sudan.

Some participants of KIIs in CAR and South Sudan identified having an official workshop or meeting with the communities to discuss the project scope to clarify expectations and effective community engagement to ensure that the communities are engaged throughout the implementation process.

### Collaboration and negotiating safe passage

Agility in service delivery was improved by working closely with local health authorities, including Health Facility Staff, local Red Cross societies/committees, the community leaders and representatives, and military authority/armed groups of respective areas for project implementation. *“..discussing with the unarmed men to show them the advantage of the programs, how it helps their families, their wives not have to give birth at home”-* Key informant, CAR.

*“They should be [able to] disseminate [information] for military about ICRC works and their views, and also let them train more volunteers by give them skills and knowledge.”-* Community leaders group South Sudan.

Collaborating with the Ministry of Health in project orientation and reporting is essential as they may often face access constraints to conflict-affected areas, whereas ICRC can access them. Safe passage was identified as essential to continue the work in the area. The participants emphasized the need to inform all political elements, including armed groups, about the planned activities, including any assessments. Crossing checkpoints was fraught with unknown dangers. The passage is safer if all the factions are aware of the activities and the implementers, CHW supervisors and CHWs are provided with proper legal documents.

### Comprehensive delivery of services within communities

Communities emphasized the need to identify with one trustworthy service provider for all their needs. This was viewed as being more acceptable and allowing NGOs to be responsible for the comprehensive delivery of services in one area, such as healthcare. Rather than fragmenting services between NGOs in one community, it will be easier for the community to connect with one organization delivering all the health and related services. Having CHWs embedded in the community with adequate supplies is vital in conflict-affected settings as war may ensue at any time and communities are blocked. With proper resources, CHWs within the communities can provide the necessary support and care during an acute outbreak of conflict. *“…[availability of] emergency box drugs so in case if there is anything bad [war/conflict] happened we may go with our treatment” –* Women’s group South Sudan.

Another aspect of enhancing the agility of health service delivery was setting up a well-structured referral system for referrals from the community to the health facilities. The participants identified that reinforcing community relay capacity was essential in conflict settings, especially when the populations are displaced due to the conflict.

### Logistical considerations to improve access

Distances and transportation emerged as crucial barriers to accessing CAR facilities. However, more importantly, this was a challenge for CHWs and their supervisors to reach the communities to deliver their services. Providing CHWs and their supervisors with bicycles was identified as a solution to distance, lack of transportation, and poor road infrastructure. The participants also identified weatherproof resources such as raincoats, boots, and rainproof bags to protect learning material as essential, especially during the rainy season. The supervisors suggested that their area of supervision should be divided into smaller manageable areas, so access to CHWs in their assigned areas is manageable. Better coordination between NGOs to set training times and avoiding any sessions during rainy and harvest seasons were likely to improve CHW participation in the training process.

### Bridging knowledge and communication gaps

All the participants agreed that the training and services should be provided in local languages, and use of innovative approaches such as use of pictures and figures to train CHWs and educate the communities was identified as a solution. To build awareness in the communities, health workers can engage in discussions in the local language and explain health-related concepts by breaking them down to easily understandable and relatable ideas. Using easy-to-understand mini-modules and methods to repeat the information multiple times was suggested to train CHWs.

### Empowering women and adolescents

Identifying women leaders with the support of the communities, setting up training that does not impact their daily lives, and support networks for women were identified as necessary to improve mother and child health in these conflict-affected communities. Engaging men to educate them about the importance of empowering women to participate as CHWs, supervisors, and leaders in delivering primary health services was essential in ensuring that women are part of the process.

There was consensus among adolescent focus groups, both boys and girls, in expressing the need to be included in assessments and wanted to be trained as CHWs to support their peers and communities.

### Resources and incentives

The issue of financial incentives was where we found discordance among the participants. Better financial incentives were identified as a solution to lack of motivation, especially those stemming from competing priorities. Program implementers and some community leaders viewed financial incentives as a demotivator for community-based work. From program implementers point of view, this work was based on charitable and innate goodwill for their communities, however, the CHWs who provided these services viewed this as extra work. Despite intending to do good for their communities, they needed to find livelihood to ensure they could provide a decent means to their families. There was consensus on the need for more resources such as medicines, basic first aid, and training materials were needed.

### Training and awareness

Participants in CAR raised the importance of working modalities to transfer knowledge to local community members to ensure service continuation and sustainability. Another strategy to improve program agility is to provide ongoing training responsive to community needs, conduct continued ongoing needs assessments, and adjust accordingly. It is essential to ensure that regular training takes place during peacetime so the CHWs are ready to respond, and all stakeholders are aware of the activities. *“..we need to talk and motivate others [and] agencies during peacetime to train and disseminate their roles and view to the military side to reduce suspected rumors and to know the importance of these services”* – CHW group South Sudan.

Additional strategies identified by the participants were regular refreshers for CHWs and supervisors in partnership with the government, engaging monitors in planning activities, including supervision-related activities and training for data monitoring, highlighting the importance of using data in planning. *“Lack of qualified or experienced community health workers is a major gap… The strategies will [entail] going back [partnering with] to the government [to set up trainings in partnership with the government]. If people are trained, they can help communities” –* Key informant, South Sudan.

Engaging community members and using unique and innovative approaches, such as radio communications and local means of arts and expression to set up awareness campaigns, is another way to improve responsiveness and agility in delivering health services. Awareness campaigns focusing on promoting messaging against Gender-Based Violence (GBV) and other health messages through the engagement of women and youth are likely to improve community responsiveness. *“…we need to be engaged because we need for us health workers from women and from youth and from boys and girls in our community” [CHWs living in community] will do it”-* Women’s group South Sudan.

## Discussion

Our respondents reported that delivering community-based RMNCAH services in areas experiencing armed conflict was not only feasible, but essential since populations’ ability to access traditional pathway of seeking health care, such as health facilities, is severely limited during times of active and/or protracted conflict. The feasibility of delivering care in these settings requires community engagement, specifically engagement of trusted community leaders and elders. It further requires that care be delivered through CHWs that are well-trained and either members of or trusted by the communities which they served. The importance of these factors in ensuring feasibility and success of program implementation emphasizes trust building at the core of delivering health services in areas experiencing armed conflict.

This study identified barriers to implementation while also providing recommendations from the field for practical, context-responsive solutions to address the barriers. Program implementers, CHW and volunteers, community leaders, and community members consistently reported similar categories of barriers. The identified barriers are not unique to CAR or South Sudan. Similar barriers to community-based health services delivery in several conflict-affect areas have been identified in numerous countries, including Afghanistan, the Democratic Republic of Congo, Kenya, Mali, Myanmar/Burma, Nigeria, Pakistan, Uganda, Yemen [18–25]. For example, low general literacy levels, and lack of understanding of health issues and basic health literacy were barriers when examining beliefs and practices around maternal and infant nutrition in Northwest Pakistan [18] and the use and uptake of maternal, newborn and child health services in Kenya [19]. Distance and road infrastructure as barriers to accessing these health services have also been identified in Afghanistan and Kenya [19, 20]. Challenges with a lack of skilled healthcare providers have been documented in Afghanistan and Uganda where insufficient technical training has hampered the ability to provide the needed health services [20, 21]. Resource scarcity and lack of supplies at health facilities during times of conflict are prominent barriers identified in this study and examined at length in Afghanistan and Myanmar [20, 22]. The role of cultural practices and the persistent role of patriarchal and the disadvantaged position of women in society as barriers that have wide-reaching impacts on RMNCAH services delivered during the conflict have been reported in Pakistan and Uganda [18, 21]. Finally, across conflict-affected countries insecurity, and resulting impacts such as restricted movement are consistent highlights as among the most significant and challenging barriers to overcome [19, 20, 23–25].

While this study identified numerous barriers to RMNACH health services delivery in the conflict-affected area, our findings demonstrate that an integrated community-based approach with sustained community engagement throughout implementation can be a feasible approach to services delivery and community uptake. Our respondents emphasized the great need for community-based health services in conflict-affected contexts and highlighted key gaps in program delivery. Our findings are consistent with evidence suggesting that planning for continuity of care is crucial when planning for health services, especially in fragile and conflict-affected settings [3, 5, 6, 26]. Respondents further reported the importance of community engagement as a facilitator of program implementation to gain communities’ trust and facilitate access to some of the most vulnerable populations that have been displaced by ongoing conflict. Interestingly, respondents also proposed feasible, community-led solutions to support RMNCAH health services delivery to populations living in conflict-affected contexts.

While systemic and structural solutions are required to support increased security and safety of populations in conflict-affected contexts, long-term strategies are more complex. Our respondents identified strategies that may be feasibly implemented in the shorter term with support from global humanitarian organizations like the ICRC and CRC. One example supported in the literature is providing regular training that is responsive to community needs [18, 22, 26]. Building on community awareness is another strategy the respondents proposed that has significant support in the literature, ensuring community engagement and leadership throughout the intervention process from planning to implementation and program evaluation [18, 24]. Furthermore, respondents suggested increasing community and stakeholder awareness by developing education and awareness campaigns that engage community members and use unique and innovative approaches, such as radio communications [21, 27, 28].

### Limitations

COVID-19 restrictions limited the ability of the researchers to access the study population to carry out focus group discussions directly. This may have hampered the understanding and/or influenced the meaning of the questions being asked and led to inconsistencies with translations and transcription verifications. However, we attempted to mitigate these challenges by engaging in an in-depth training-of-trainers process to equip focus group facilitators with the necessary skills and tools to execute the focus groups discussions effectively, in local language by those who were trusted by the communities. Since the interviews were conducted by locals, by those who understood the contexts, they were also able to pick up nuances and direct more appropriate follow up. Additional information was ascertained through the KII with program implementers to fill the gaps and omissions.

## Conclusion

Inclusive, contextualized, and localized service delivery, as delineated in the APiH framework can serve as feasible modality for delivery of health services in conflict affected areas. While.security and trust related issues, knowledge and communications gaps, logistical issues and lack of motivation remain barriers to delivery of health services especially in conflict settings; using an integrated public health approach with core activities integrated with context specific services guided by community consultations can support delivery of health services in these contexts.

To achieve successful, agile, and responsive implementation of community-based health services in a conflict-affected setting, decision-makers and program implementers should focus on engaging communities throughout the life cycle of the program, take into account services based on community needs where possible, ensure all relevant stakeholders in armed conflict are approached for engagement, bridge inequities through the engagement of vulnerable groups, such as women and adolescents, negotiate safe passage for healthcare workers, keep logistical and resource constraints under consideration and contextualize service delivery with the support of local actors. Our study contributes to the global literature on effective health services in conflict-affected areas and provides operational insights for decision-makers, program implementers and community members.

## Supplementary Information

**Additional file 1.**

**Additional file 2.**

## Data Availability

The datasets generated and/or analyzed during the current study are not publicly available but may be available from the corresponding author on reasonable request.

## Abbreviations

APiH: Advanced Partnership in Health
BHW: Boma health worker
CAR: Central African Republic
CHWs: Community health workers
COVID-19: Coronavirus disease of 2019
CRC: Canadian Red Cross
CRCA: Croix-Rouge centrafricaine
FG: Focus Group Discussions
GBV: Gender-based violence
ICRC: International Committee of the Red Cross
IFRC: International Federation of Red Cross and Red Crescent Societies
KII: Key informant interviews
MoH: Ministry of Health
HNS: Host National Societies
PSEA: Prevention and Response to Sexual Exploitation and Abuse
PSS: Psychosocial Support
RMNCAH: Reproductive, Maternal, Neonatal, Child, and Adolescent Health
SSRC: South Sudan Red Cross
ToT: Training of Trainers
WHO: World Health Organization

## Footnotes

1 Details about the International Committee of the Red Cross mandate, history and areas of work can be found on their website: https://www.icrc.org/en. Accessed last on April 17^th^ 2023.

2 Advanced Partnership in Health framework co-designed by ICRC and CRC defined Integrated packages of services: as much as possible, the community-based RMNCAH services will be delivered as packages to maximize their reach taking advantage of existing program and services to deliver life saving interventions to mother, children and adolescents. RMNCAH Framework_Advanced Partnership_ICRC & PNS_ Geneva_ 2018. Sigrid Kopp, Juliane Boucquiaux ICRC Mariam Kone, Canadian Red Cross.

3 In the communities in South Sudan, it was easier to find CHWs as most of those had been identified through the government’s Boma Health Initiative Policy. Boma Health Workers or BHWs a network of volunteers organized by the Ministry of Health in South Sudan works in communities to bridge the gap between community and health facilities. The network consists of the Boma Health Committee, the Boma health workers, and the Boma health supervisors.
