# Supplementary material for: Implementing community-based health program in conflict settings: documenting experiences from the Central African Republic and South Sudan
Source: BMC Health Serv Res. 2023 Jul 8;23:738. doi: 10.1186/s12913-023-09733-9 (PMC10329391; doi:10.1186/s12913-023-09733-9)
Supplement: Supplementary file 1 — Additional file 1. [file 12913_2023_9733_MOESM1_ESM.pdf]

SUPPLEMENT 1: Interview guides for Key Informants and Focus Groups

1. Semi-Structured Interview Guide: Program Implementers

APiH Proof of Concept

Expected participants:

Multiple stakeholders- project officers, supervisors, project leads, policy makers in both countries. These stakeholders will consist of various implementers engaged in the implementation of the project, from field to those working at headquarters in South Sudan and CAR to those in Ottawa and in Geneva working to implement the project. Expected to interview 12-16 participants in total from South Sudan, CARC, CRC and ICRC.

Preamble/Project introduction blub:

We have a lot of ground to cover. We have 8 themes to address and hope to finish the interview within 45-1hour.

Interview guide:

Semi structured and open-ended guide (data collectors are expected to explore) Starting questions was their general opinion about the APiH program and was it working in their communities.

Theme 1: Opportunities for, challenges and solutions to community-based assessments (e.g. baseline needs assessments)- (South Sudan, CAR, CRC, ICRC)

Suggested questions

1. Can you tell me about conducting community-based assessments during conflicts?

[Probe]

- Is possible/feasible to conduct these assessments?
  - Why or why not?

2. From your perspective as a program implementer, what are some barriers and challenges to conducting these assessments?

3. From your perspective as a program implementer, what are some potential solutions to address the *barriers/challenges* [use this opportunity to repeat and reinforce the barriers identified by the respondent] you have identified?

Theme 2: Opportunities for, challenges and solutions to safe and responsible engagement of the elders and community leaders - (South Sudan, CAR, CRC, ICRC)

Suggested questions

1. Can you please describe experiences that you have had engaging the community in conflict settings?

[Probe]

- In particular, could you highlight the role of elders and community leaders in this engagement?

2. What were some of the key challenges you face in your attempts to engage the community in the examples [use this opportunity to repeat and reinforce the experiences described by the respondent] you provided?

3. How do you believe these key challenges [use this opportunity to repeat and reinforce the challenges/barriers described by the respondent] could be remedied in the future?

Theme 3: Opportunities for, challenges and solutions to identification and recruitment of Community Health Workers (CHWs)/Volunteers- (South Sudan, CAR)

Preamble: The next series of questions I will ask you specifically relate to the role of community health workers and volunteers specifically regarding recruitment, training, delivery of health services, and supervision.

Suggested questions

1. Firstly, please describe the processes used to recruit CHWs and/or volunteers?

[Probes]

- How are potential recruits identified?
- What skills or experiences are required?
- other
- Do you find there are any barriers that arise or challenges you are faced with when attempting to recruit these individuals?
    - Can you please elaborate/explain?

2. What do you think are some key strategies that can be to mitigate/address these challenges [use this opportunity to repeat and reinforce the challenges/barriers described by the respondent]?

Theme 4: Opportunities for, challenges and solutions to training of Community Health Workers (CHWs)/Volunteers- (South Sudan, CAR)

Suggested questions

1. What is your understanding of the training processes for CHWs/volunteers?

[Probes]

- What are some of the specific skills that they are trained on?
- Where does the training occur?
- How long are the training session?
- How many training sessions occur prior to the initiation of work?
- What is involved in the training session?
- Can you describe a typical training session that you have been a part of?

2. Can you please describe current/ongoing trainings for this project?
    a. If there are current trainings:
        i. What do you find are some of the key difficulties when engaging in training sessions with CHWs/volunteers?
        ii. How do you believe some of these barriers can be overcome?
    b. If trainings have not yet begun:
        i. Where are you at with the planning process?
        ii. Why haven't the trainings not begun?
        iii. What are some of the challenges?
            1. To beginning?
            2. Anticipated challenges when you start?

Theme 5: Opportunities for, challenges and solutions to delivery of health services by Community Health Workers (CHWs)/Volunteers- (South Sudan, CAR)

Suggested questions

1. How do CHWs/volunteers deliver services to the community?
2. What are some of the barriers and challenges?
3. What do you believe are possible solutions to the challenges you have identified?

Theme 6: Opportunities for, challenges and solutions to Supervision of Community Health Workers (CHWs) (CHWs)/Volunteers- (South Sudan, CAR)

Suggested questions

1. Can you describe what is involved in the process of supervising CHW/volunteers?

[Probe]

- Have you been responsible for supervising CHW/volunteers?
- What were some of the major challenges you faced?

3. How can the process of supervision be facilitated to better support the supervisor and the CHWs/volunteers?

Theme 7: Opportunities for, challenges and solutions to monitoring- (South Sudan, CAR, CRC, ICRC)

Preamble: Moving forward from our focus on CHWs/volunteers, I will now ask you some questions about program monitoring.

Suggested questions

1. Can you please describe some of the existing mechanisms for program monitoring?

[Probes]

- Who is responsible for monitor?
- How does monitoring take place?
- What do you find are some of the biggest challenges involved in monitoring program implementation in [name of context]?
- How do you propose these challenges could be remedied?

Theme 8: Improving agility of community-based health services in conflict settings South Sudan, CAR, CRC, ICRC)

1. Could you highlight what you believe are the major gaps in delivery of community-based health services in conflict settings?

2. What strategies to you think could be employed to improve the responsiveness of programming community-based health services programming to conflict settings?

Closing Questions:

Is there anything else that you wish to add?

Would you be comfortable being contacted for follow-up when the project has been implemented?

2. Focus Group Discussion Guide: Beneficiaries (South Sudan & CAR)

Advanced Partnerships in Health (APiH)

Expected participants:

Two focus groups (one in each country). 6 to 10 participants per focus group with beneficiaries (12 to 20 participants in total). Ensuring groups are gender balanced and equal representation of women including mothers and also including fathers and adolescents in the group.

Interview guide:

Semi structured- open ended guide (data collectors are expected to explore)

Theme 1: Opportunities for, challenges and solutions to safe and responsible engagement of the elders and community leaders - (South Sudan, CAR, CRC, ICRC)

Suggested questions

1. Do you believe it usefulness for you and your community leaders to engage in delivery of community-based health programs?

[Probes]

- What do you believe are some of the barriers to engagement?
- How do you believe engagement can be improved? Please provide any suggestions for possible solutions to overcomes the barriers the group has identified.

Theme 2: Opportunities for, challenges and solutions to delivery of health services by (CHWs)/Volunteers- (South Sudan, CAR)

Suggested questions

1. As people who access and use of health services, can you discuss some of your experiences receiving health services that were being delivered by CHWs and/or volunteers?

[Probe]

- What are some of the challenges?
- How do you believe delivery of health services by these CHWs and volunteers can be improved?

Theme 2: Improving agility of community-based health services in conflict settings (South Sudan, CAR, CRC, ICRC)

1. Please discuss/describe some of the key shortcomings in the delivery of health services in your community?

2. Considering your context, how do you believe the response to you and your community's health needs and delivery of health services can be improved?

3. Focus Group Discussion Guide: CHWs and Volunteers (South Sudan & CAR)

Advanced Partnerships in Health (APiH)

Expected participants:

Four focus groups (one for CHWs and one for volunteers in both countries). Each group will be comprised of 6-10 participants. A total of 20 to 24 respondents will participate. Ensuring groups are gender balanced and equal representation from female CHWs and Volunteers.

Interview guide:

Semi structured- open ended guide (data collectors are expected to explore)

Theme 1: Opportunities for, challenges and solutions to training of Community Health Workers (CHWs)/Volunteers- (South Sudan, CAR)

Suggested questions

1. Can you please describe the processes used to train you to delivery health care services to your community?
2. What did you find were some of the most significant challenges you faced when receiving training?
3. How do you believe these challenges could be overcome?

[Probe]

How can training better support you to deliver health services in your community?

Theme 2: Opportunities for, challenges and solutions to delivery of health services by (CHWs)/Volunteers- (South Sudan, CAR)

Suggested questions

1. Please describe how you participate in delivering health services to the community?

Exploring

2. Do you find that you have adequate supplies to carry out your duties? Explain/Elaborate.

[Probe]

- When supplies require replenishing, what process do you use to get more supplies?
  - Who do you contact? Who is responsible?
  - Where does the funding come from?

Exploring the issues of equipping CHWS with adequate supplies and renewal of supplies

3. Beyond supplies, what are some other key challenges face by CHWs and volunteers like yourself when delivering in health services in the community?

4. What are some recommendation you would make to overcome some of the challenges the group has identified?

Theme 3: Opportunities for, challenges and solutions to Supervision of (CHWs)/Volunteers- (South Sudan, CAR)

Suggested questions

1. Can you tell us about how your work has CHWs and volunteers is supervised?

[Probe]

- Who is responsible for your supervision? Who do you report to?

2. What are some of the key challenges?

3. How the identified challenges be remedied?

Theme 4: Opportunities for, challenges and solutions to monitoring- (South Sudan, CAR, CRC, ICRC)

Suggested questions

1. Can you please discuss the processes in place for monitoring the delivery of health services in your community?
2. What do you believe are some of the challenges or barriers to effective monitoring?
3. What are your recommendations for addressing the identified challenges?

Theme 5: Improving agility of community-based health services in conflict settings South Sudan, CAR, CRC, ICRC)

1. Can you discuss some of the gaps in delivery of community-based health services in conflict settings?
2. How might the swiftness of the response and delivery of health services be improved in your communities/in conflict settings?

4. Focus Group Discussion Guide: Village Elders and Community Leaders (South Sudan & CAR)

Advanced Partnerships in Health (APiH)

Expected participants:

Two focus groups (one in each country). 6 to 10 participants per focus group- 12 to 20 participants in total) with village elders and community leaders. Ensuring that village elders are included in each group and the groups are gender balanced.

Interview guide:

Semi structured- open ended guide (data collectors are expected to explore)

Theme 1: Opportunities for, challenges, and solutions to community-based assessments (e.g., baseline needs assessments)- (South Sudan, CAR, CRC, ICRC)

Suggested questions

1. Is it possible to conduct community-based assessments during conflicts (if yes- how? If no- why)
2. Exploring barriers and challenges for assessments
3. Exploring possible solutions

Theme 2: Opportunities for, challenges and solutions to safe and responsible engagement of the elders and community leaders - (South Sudan, CAR, CRC, ICRC)

Suggested questions

1. Can you describe your experiences as an elder/community leader when supporting the delivery of health services within your communities?

[Probes]

- Are there any barriers to your safe engagement?
- How do you believe these barriers can be overcome?

Theme 8: Improving agility of community-based health services in conflict settings South Sudan, CAR, CRC, ICRC)

1. What are some of the major gaps in the delivery of community-based health services in conflict settings?
2. How can delivery of health services in conflict settings be improved?
