# Supplementary material for: Implementing community-based health program in conflict settings: documenting experiences from the Central African Republic and South Sudan
Source: BMC Health Serv Res. 2023 Jul 8;23:738. doi: 10.1186/s12913-023-09733-9 (PMC10329391; doi:10.1186/s12913-023-09733-9)
Supplement: Supplementary file 2 — Additional file 2. [file 12913_2023_9733_MOESM2_ESM.pdf]

Supplement 2: Codebook developed based on three study objectives

Objective 1: Perspectives on feasibility (factors contributing to feasibility of services)

Theme	Codes	Description	Example Quotes
**Factors contributing to feasibility of services**	Clear messaging	Clear communication using easy-to-understand materials in local languages to gain community trust and buy-in	"If clear message is delivered and they understand the situation and they know what is going to take place... they will accept." - Program implementer, South Sudan
	Inclusive participation	Involving community leaders and members in the planning, design, and implementation of services	"Chiefs were there [community engagement and assessment sessions] so that provide legitimacy." - Program implementer, South Sudan
	Community delivery	Selecting community healthcare worker trainees who are native to the community and committed to staying with the community during conflict	"…we need for us health workers from women and from youth and from boys and girls in our community to learn about the work for the next time these people cant come around [because of war] they [CHWs living in community] will do it." - Women's group, South Sudan

Objective 2: Barriers to implementation of community-based RMNCAH services in conflict-affected areas

Theme	Category	Codes	Description	Example Quotes
Barriers to implementation of community-based RMNCAH services in conflict-affected areas	Security	Security_active conflict<br>Security_isolated communities<br>Security_government reach	Hindrance to program implementation due to insecurity and physical barriers to access communities isolated in conflict areas	"In our context the biggest challenge is security, I don't think we have a bigger challenge than that" - Program Implementer, CAR.
	Trust	Trust_implementation process<br>Trust_past assessments<br>Trust_non-natives	Lack of trust in past assessments and mistrust of non-natives hindering implementation process	"We have participated in this kind of assessment many times and there is need to follow up on the assessment results for the success of the community members" - Community leaders group, South Sudan.
	Resources	Resources_medicines<br>Resources_human resources<br>Resoucres_referral pathways<br>Resources_tools	Resource scarcity affecting implementation of services	"problem is a when you give awareness to somebody in the community, and then you send that person for- for services and that service happens not to be there" -

			Program implementer, South Sudan
Access	Access_poor weather<br>Access_poor road conditions<br>Access_long distances<br>Access_mobility related issues	Mobility-related issues, especially for women and children, affecting access to health services	"...the distance or the road also matters. Like here in our in our context, there are other places where by bicycle cannot go. [In other areas] vehicles cannot go unless maybe bicycles or maybe sometimes on foot and the other thing is somebody who might not know how to ride a bicycle it can still be a barrier. " - Program implementer, South Sudan
Motivation	Motivation_inadequate recruitment<br>Motivation_competing priorities<br>Motivation_youth engagement<br>Motivation_delays in service delivery or implementation of programs	Lack of motivation	"..someone having responsibility at home they need to go for long period time. People are dependent on others to get daily meal so that would be difficult"- Program implementer, South Sudan

	Knowledge	Knowledge_language barriers<br>Knowledge_health literacy<br>Knowledge_miscommunication about available services	Knowledge and communication gaps	"Most of them they don't write. We need to continually recap to make them remember what they have...Handling materials is a challenge in rainy season as their materials can get wet. ...Language barriers can be an issue, that is a big one." - Program implementer, South Sudan
Barriers to identification, recruitment, training, and supervision of CHWs	Socio-cultural	Socio-cultural_competing family responsibilities<br>Socio-cultural_women dropping out of training<br>Socio-cultural_husband's approval	Traditional beliefs and practices which can conflict with recruitment, training, and deployment of CHWs	"sometimes when you select a woman who is the at home , of course we need responsible people. They will say they first need to contact their husband for approval to accept. "- Program implementer, South Sudan
	Recruitment	Recruitment_women's participation<br>Recruitment_inequitable recruitment<br>Recruitment_availability<br>Recruitment_farming season	Factors which lead to non-representative recruitment of CHWs	"In some communities...a chief will be influenced...to bring all his sons and daughters. So, this is when normally we come in and we start doing verification of the names, then automatically look and will say no. We cannot bring all volunteers from the same family. Not all

			the same house" - Program implementer, South Sudan
Logistics	Logistics_long distances services to households<br>Logistics_Inadequate supplies during rainy season<br>Logistics_loss of training materials	Logistical factors which impact the recruitment, tranining, or deployment of CHWs	"We need to arrange for fuel, car, and only the far distance is the challenge." Program implementer, South Sudan
Communication gaps	Communication_Lack of overall literacy<br>Communication_Lack of data literacy<br>Communication_Miscommunications about available services<br>Communication_Language barriers	Gaps in communication channels and low awareness of health services	"Literacy is an issue overall in SS. Some can read and write and some can understand if it's explained to them and carryout their activiites. Some are bit educated some not. Those who are not educated but can get proper explanationd and but can bring the message they are ok- They key thing is to understand health issues, they will need to understand cases and can manage using tools at community level" - Program implementer, South Sudan

Objective 3: Strategies for context specific agile and responsive programing in conflict settings

Theme	Codes	Description	Example quotes
Strategies for context specific agile and responsive programing	Community engagement and leadership	Engaging community leaders and other stakeholders to ensure community responsiveness	“Chiefs were there [community engagement and assessment sessions] so that provide legitimacy” – Key informant, South Sudan
	Collaboration and negotiating safe passage	Working closely with local health authorities, including Health Facility Staff, local Red Cross societies/committees, the community leaders and representatives, and military authority/armed groups of respective areas for project implementation	"…discussing with the unarmed men to show them the advantages of this project” – Key informant, CAR.
	Comprehensive delivery of services	Providing comprehensive healthcare services through one trustworthy service provider	“…[availability of] emergency box drugs so in case if there is anything bad [war/conflict] happened we may go with our treatment” – Women’s group, South Sudan.
	Logistical considerations	Addressing transportation barriers and ensuring CHW access to communities to improve access	"...to overcome we give them bicycles to make sure they are able to go from BHW to another." - Program implementer, South sudan
	Bridging knowledge and communication gaps	Providing training and services in local languages, engaging in local languages, and using innovative approaches such as pictures and figures in communicaiton materials.	" Language barrier was a challeng as they speak in different dialects. They understand Arabic but feelings are expressed in local language. So hired people with capacity in local dialect and understanding of the project" - Program implementer, South Sudan

Empowering women and adolescents	Actively involving women leaders and engaging adolescents in healthcare delivery	"South Sudan is [a] patriarchal community, men leader of the household. Engaging women was our agenda, was difficult. Over time with dialogue and positioning the project as women focused- convinced that having women was important but let them know that men's engagement was important. Over time with counceling they came around and now in Godakala and Goku BHW 50% in committee and over 70% CHWs would be women. It took 3 to 4 months in regular meetings and visits to inform what value women would add" - Program Implementer, South Sudan
Resources and incentives	Providing financial and non-financial incentives for CHWs	"... The problem is ... it is difficult to leave home without daily wage. If they [CHWs] are getting some motivation they will go" - Program implementer, South Sudan
Training and awareness	Providing ongoing training responsive to community needs, conducting continued ongoing needs assessments, and adjusting the training accordingly	“we need to talk and motivate others [and] agencies during peacetime to train and disseminate their roles and view to the military side to reduce suspected rumors and to know the importance of these services” – CHW group, South Sudan
